# A Host Cell Vector Model for Analyzing Viral Protective Antigens and Host Immunity

**DOI:** 10.3390/ijms26157492

**Published:** 2025-08-02

**Authors:** Sun-Min Ahn, Jin-Ha Song, Seung-Eun Son, Ho-Won Kim, Gun Kim, Seung-Min Hong, Kang-Seuk Choi, Hyuk-Joon Kwon

**Affiliations:** 1 Laboratory of Poultry Medicine, Department of Farm Animal Medicine, College of Veterinary Medicine and BK21 PLUS for Veterinary Science, Seoul National University, Seoul 08826, Republic of Korea; vicky.ahn@snu.ac.kr; 2 Research Institute for Veterinary Science, College of Veterinary Medicine, Seoul National University, Seoul 08826, Republic of Korea; sjh1243@snu.ac.kr (J.-H.S.); arbre04@snu.ac.kr (S.-E.S.); iamkhw52@snu.ac.kr (H.-W.K.); smilesssss@snu.ac.kr (G.K.); topkin@snu.ac.kr (S.-M.H.); 3 Laboratory of Avian Diseases, College of Veterinary Medicine, Seoul National University, Seoul 08826, Republic of Korea; 4 Laboratory of Veterinary Pharmacology, College of Veterinary Medicine, Research Institute for Veterinary Science, Seoul National University, Seoul 88026, Republic of Korea; 5 GeNiner Inc., Seoul 08826, Republic of Korea

**Keywords:** vaccine, influenza A virus, humoral immunity, CD8+ T cell epitope, chicken, cell vector

## Abstract

Avian influenza A viruses (IAVs) pose a persistent threat to the poultry industry, causing substantial economic losses. Although traditional vaccines have helped reduce the disease burden, they typically rely on multivalent antigens, emphasize humoral immunity, and require intensive production. This study aimed to establish a genetically matched host–cell system to evaluate antigen-specific immune responses and identify conserved CD8+ T cell epitopes in avian influenza viruses. To this end, we developed an MHC class I genotype (B21)-matched host (Lohmann VALO SPF chicken) and cell vector (DF-1 cell line) model. DF-1 cells were engineered to express the hemagglutinin (HA) gene of clade 2.3.4.4b H5N1 either transiently or stably, and to stably express the matrix 1 (M1) and nucleoprotein (NP) genes of A/chicken/South Korea/SL20/2020 (H9N2, Y280-lineage). Following prime-boost immunization with HA-expressing DF-1 cells, only live cells induced strong hemagglutination inhibition (HI) and virus-neutralizing (VN) antibody titers in haplotype-matched chickens. Importantly, immunization with DF-1 cells transiently expressing NP induced stronger IFN-γ production than those expressing M1, demonstrating the platform’s potential for differentiating antigen-specific cellular responses. CD8+ T cell epitope mapping by mass spectrometry identified one distinct MHC class I-bound peptide from each of the HA-, M1-, and NP-expressing DF-1 cell lines. Notably, the identified HA epitope was conserved in 97.6% of H5-subtype IAVs, and the NP epitope in 98.5% of pan-subtype IAVs. These findings highlight the platform’s utility for antigen dissection and rational vaccine design. While limited by MHC compatibility, this approach enables identification of naturally presented epitopes and provides insight into conserved, functionally constrained viral targets.

## 1. Introduction

Due to the rising global demand for poultry products, the global chicken population was estimated to have reached approximately 27.22 billion in 2023 [1], a figure that notably exceeds that of other domestic animals. However, the poultry industry is currently experiencing significant economic losses from various microbial pathogens, including recent severe outbreaks of highly pathogenic H5N1 avian influenza A viruses (IAVs). Therefore, developing cost-effective and efficacious vaccines is urgently needed [2]. In this context, identifying highly protective antigens that elicit both humoral and cellular immune responses is crucial for vaccine development.

Various vaccine platforms have been explored to achieve this goal, each with distinct advantages and limitations. In addition to conventional approaches, multigenic vaccines, such as whole virions, have been widely used. However, these vaccines complicate the assessment of individual gene or mutational contributions to immunity and vaccine efficacy [3]. In contrast, monogenic vaccines, including DNA and recombinant protein vaccines, provide precise insights into immune responses induced by a single antigen, despite their limitations. DNA vaccines are simple to produce and stimulate both humoral and cellular immunity, but often exhibit low, inconsistent immunogenicity [4,5]. Recombinant protein vaccines, on the other hand, require complex gene expression and protein purification processes and primarily induce humoral immunity.

To overcome these limitations, RNA-based vaccine technologies have rapidly advanced, with their efficacy and versatility notably demonstrated during the COVID-19 pandemic [6]. Among these, mRNA vaccines have emerged as a particularly promising platform, offering advantages such as controlled production and high protein expression, although challenges remain regarding their stability, the immunogenicity of delivery components, and long-term safety [7].

While each platform offers distinct advantages, their limitations highlight an ongoing need for alternative strategies. To address this need, a non-tumorigenic cell line with high transfection efficiency and tolerance for viral gene expression represents an ideal cell vaccine platform. Moreover, a host animal compatible with this cell vector and possessing a well-characterized immune genetic background offers valuable support for experimental studies evaluating viral protective antigens and host immune responses.

Chickens serve as an essential model for vaccine research due to the availability of outbred specific pathogen-free (SPF) populations and diverse infection models. Their single dominantly expressed MHC class I molecules (B haplotypes) play a key role in modulating pathogen responses and simplifying data analysis [8]. Moreover, in this study, the use of chickens was further justified by the need to ensure MHC compatibility between antigen-presenting DF-1 cells and the host immune system. The avian MHC is structurally more compact and genetically streamlined compared to its mammalian counterpart, facilitating clearer interpretation of T cell immune responses and epitope presentation.

In this study, we established a B21 haplotype-based DF-1 cell line vector and VALO SPF chicken models to analyze viral protective antigens and host immunity Specifically, humoral immunity was assessed through the immune response to hemagglutinin (HA), while cellular immunity was evaluated by measuring IFN-γ production from peripheral blood mononuclear cells (PBMCs) following immunization with DF-1 cells expressing matrix 1 (M1) or nucleocapsid protein (NP). Additionally, potential CD8+ T cell epitopes were identified within HA, M1, and NP. Our findings validate our host cell vector system as a tool for evaluating protective viral antigens and host immune responses, with implications for designing next-generation vaccines.

## 2. Results

### 2.1. DF-1 Cell Lines Are Adequate for Efficient Expression of Transient and Permanent IAV Genes

**Figure 1:**
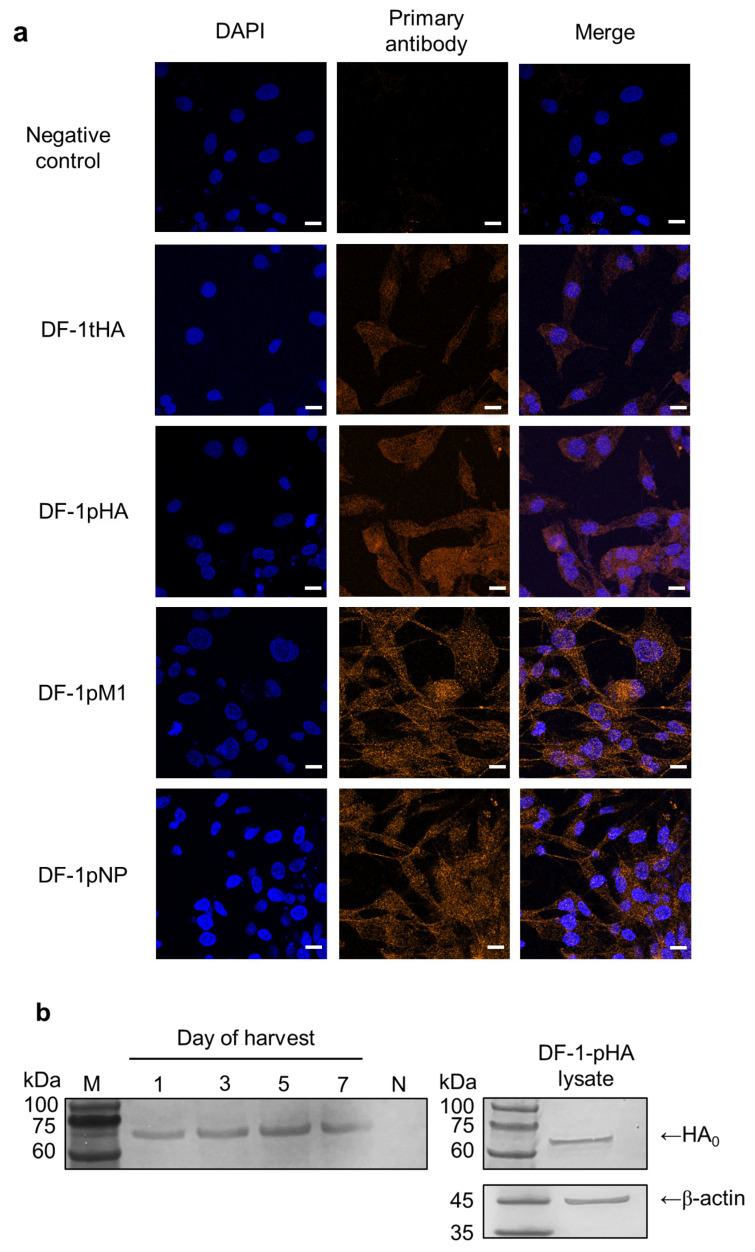
DF-1 cells transiently or permanently expressing the HA gene of 2.3.4.4b H5N1 and M1 and NP genes of SL20. DF-1-tHA was prepared via transfection with the pcDNA3.1_HA expression vector using Lipofectamine 2000, and DF-1-pHA, DF-1-pM1, and DF-1-pNP were established using the lentiviral expression system. The scale bar (10 µm) is indicated by a white line. (**a**) Confocal imaging of DF-1 cells transiently or permanently expressing the null (negative), HA, M1, and NP genes. (**b**) Western blot analysis of culture supernatants collected at different time points demonstrating the presence of HA protein. Prominent HA protein bands were detectable starting from 1-day post-seeding. HA expression was confirmed using cell lysate as a positive control, and β-actin served as a loading control. M: protein size marker, N: supernatant from negative control DF-1 culture.

Over 90% of cells transiently expressed HA (DF-1-tHA), and confocal microscopy confirmed HA-specific signals in both transient (DF-1-tHA) and permanent (DF-1-pHA) DF-1 cells (Figure 1a). Additionally, DF-1 cell lines that permanently express the M1 (DF-1-pM1) and NP (DF-1-pNP) genes from the SL20 virus were established, and their expression levels were high enough to be detected using confocal microscopy and antiserum.

To determine whether HA proteins were secreted into the culture supernatant, supernatants from DF-1-pHA cultures were collected at 1-, 3-, 5-, and 7-days post-seeding for Western blot analysis. For comparison, DF-1-pHA cell lysates were also analyzed. Bands corresponding to HA were detected at all time points, suggesting that HA-containing exosomes may be released as early as one-day post-seeding. To reflect practical vaccine administration conditions, cell preparations were used without additional washing steps, thereby preserving vesicle-associated antigens potentially present in the culture supernatant. This approach also enabled the evaluation of unprocessed cell preparations as a simple and scalable vaccine strategy. Accordingly, both cells and their supernatants were used in subsequent experiments (Figure 1b).

Although we used a wild-type HA gene encoding polybasic amino acids at the proteolytic cleavage site, only the uncleaved HA_0_ precursor was detected in the supernatant via Western blotting, with no clear separation into HA1 and HA2 subunits. One possible explanation relates to cell-line-specific characteristics. DF-1 cells, unlike HEK293T or MDCK cells, express lower levels of endogenous furin-like proteases, limiting the cleavage of polybasic substrates such as HA_0_ [9]. It is known that fibroblast-derived cell lines, including DF-1, NRK, and MSU-1.1, express furin primarily in the trans-Golgi network where precursor protein processing occurs [10]. However, under conditions of high-level HA expression via lentiviral vectors, the available endogenous furin activity may be insufficient to process all newly synthesized HA_0_ molecules. This insufficient processing capacity likely resulted in the accumulation of uncleaved HA_0_, as observed in our Western blot analysis where the majority of supernatant HA protein appeared in the uncleaved form.

### 2.2. MHC Class 1 Molecule Haplotype of DF-1 Cells Matches with Most VALO SPF Chickens

**Figure 2:**
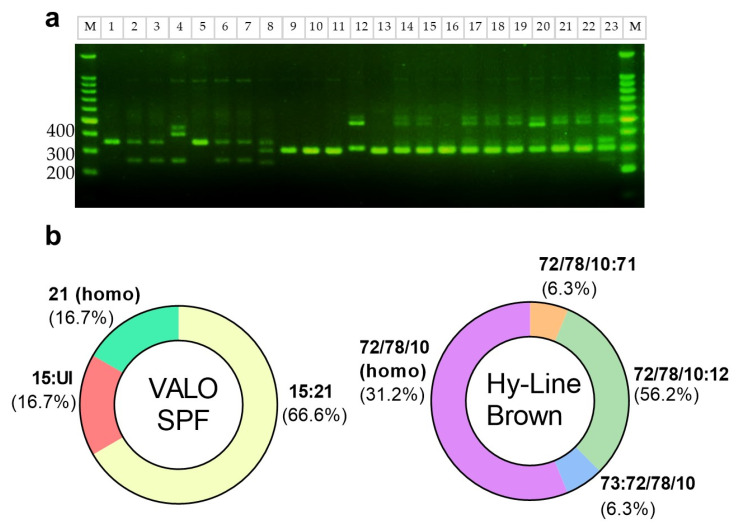
Identification of MHC class 1 haplotypes of VALO SPF and Hy-Line brown layer chickens. (**a**) LEI0258 microsatellite marker amplification and agarose gel electrophoresis. M, DNA marker; lane 1, DF-1 (homozygote B21, 357 bp); lanes 2–7, VALO SPF chickens—lanes 2, 3, 6, and 7: heterozygote B21:B15 (261 bp); lane 5: homozygote B21; lane 4: heterozygote B15: unidentified; lane 8–23, commercial chickens—B72 and B78 (307 bp), B10 (309 bp), B12 (487 bp), B71 (474 bp), and B73 (249 bp); lane 8: B73: B72/B78/B10; lanes 9–11, 13, and 16: B72/B78/B10: B72/B78/B10; lanes 12, 14, 15, 17–19, and 21–23: B72/B78/B10: B12; lane 20: B72/B78/B10: B71. (**b**) Frequency of B haplotypes in VALO SPF and Hy-Line commercial chickens.

In accordance with previous reports indicating that DF-1 cells are homozygous for the B21 MHC class I haplotype, we first confirmed this through LEI0258 tandem-repeat PCR analysis [11]. We then applied the same method to identify the MHC class I haplotypes of VALO SPF chickens and commercial Hy-Line Brown layer chickens (Figure 2a), whose haplotypes have been previously reported. In a prior study, VALO SPF chickens were shown to harbor B15:B21 (40.9%), B21:B21 (8.8%), B15:B19 (15.2%), and other haplotypes (35.9%), with corresponding amplicon sizes of 357 bp (B21), 261 bp (B15), and 539 bp (B19) [12]. Out of six VALO SPF chickens, one was homozygous for the B21 haplotype, four were heterozygous (B15:B21), and only one lacked B21 (B15:UI) (Figure 2b). Further MHC haplotype analysis (*n* = 40; electrophoresis) showed that 82.5% of the birds carried the B21 haplotype, with 30% (12/40) being homozygous and 52.5% (21/40) heterozygous. The remaining 17.5% (7/40) were non-B21. In contrast, Hy-Line Brown layer chickens exhibited band patterns indicative of both homozygosity (31.2%, 5/16) and heterozygosity (68.8%, 11/16) (Figure 2a), but none of the observed bands matched the B21 amplicon size. Although the exact haplotypes could not be conclusively identified, based on fragment size and previous reports, these bands may correspond to B72 and B78. Prior studies have shown that B10 and B78 correspond to 309 bp and 307 bp, respectively, while B12 and B71 measure 487 bp and 474 bp [13], none of which overlap with the 357 bp B21 amplicon (Figure 2b).

### 2.3. B21 Haplotype Confirmation Through Sequence-Based Typing (SBT)

**Figure 3:**
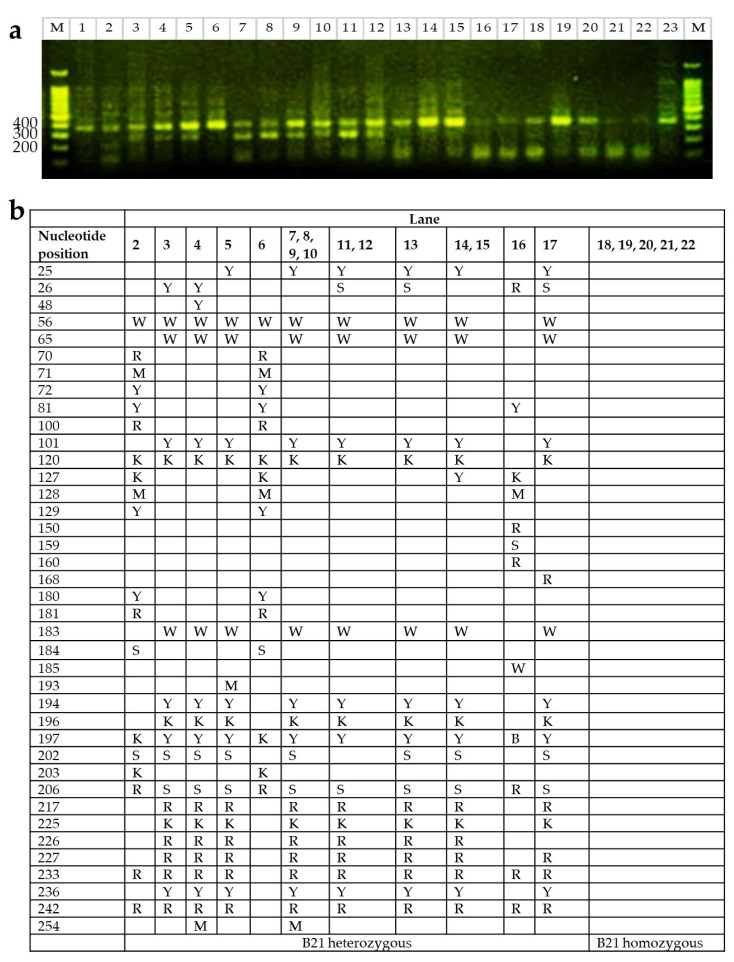
Genotypic polymorphisms and corresponding PCR banding patterns of samples. (**a**) Amplification of the LEI0258 microsatellite marker and visualization by agarose gel electrophoresis. M, DNA size marker; lane 1 and lane 23: DF-1 cell line (B21 homozygote, 357 bp); lanes 2–22, subset of individuals previously classified as B21 homozygous or heterozygous based on LEI0258 PCR band size. (**b**) Genotypic polymorphisms in the BF2 allele confirmed by Sanger sequencing. Heterozygous nucleotide positions were annotated using IUPAC codes based on overlapping peaks observed in both forward and reverse chromatograms (W = A/T, R = A/G, M = A/C, Y = C/T, K = G/T, S = G/C, B = C/G/T).

To validate B21 haplotype classification based on LEI0258 PCR banding patterns, a subset of individuals previously identified as B21 homozygous or heterozygous by PCR were selected for Sanger sequencing of the BF2 locus (Figure 3a). Among the 23 samples sequenced, five were confirmed to possess B21 homozygous sequences. The remaining samples displayed variations consistent with heterozygosity at the BF2 locus, with several sharing identical heterozygous sequences (Figure 3b).

Although it is generally acknowledged that LEI0258 tandem repeat typing alone may not be sufficient for definitive MHC haplotype determination, particularly under variable or field conditions, our sequencing results support the reliability of LEI0258-based PCR typing when applied in a controlled experimental setting. In this study, we used DF-1 cells with a known B21 haplotype as a reference to guide band interpretation, ensuring consistency and accuracy. These results demonstrate that, within well-defined laboratory conditions, LEI0258 typing can yield outcomes that are highly consistent with sequence-based haplotyping. This aligns with previous studies showing that the LEI0258 marker, due to its extensive allelic diversity, can serve as a dependable indicator of MHC haplotypes even in genetically diverse populations [11]. Accordingly, LEI0258 typing continues to be widely used in research and breeding for initial MHC genotyping and selection planning.

### 2.4. Live DF-1 Cell Expressing HA Gene Induces Strong Humoral Immunity in VALO SPF Chickens

**Figure 4:**
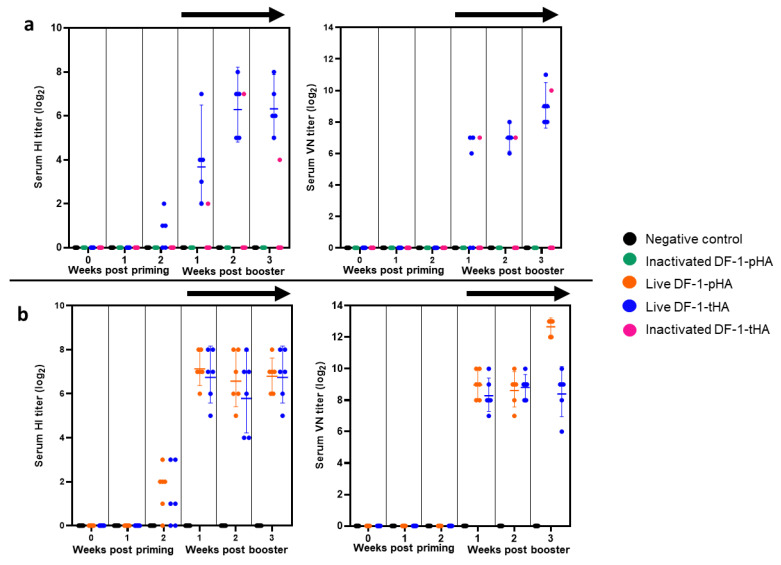
Comparison of humoral immune responses induced by DF-1 cell vaccines expressing HA genes in VALO SPF chickens. (**a**) Primary experiment: HI and VN titers were compared between live and inactivated cells expressing HA genes to assess their ability to stimulate humoral immunity. (**b**) Secondary experiment: HI and VN titers were compared following immunization with live DF-1-tHA and live DF-1-pHA vaccines. Homo- and heterozygous B21 haplotype chickens were used, and vaccines (2 × 10^6^ cells/dose/chicken) were administered via intramuscular route. The black arrow indicates measurement after booster vaccination. Geometric mean values are shown, and error bars represent the 95% confidence interval.

In the first experiment, we compared the immunogenicity of live DF-1-tHA with that of inactivated DF-1-pHA and DF-1-tHA (Figure 4a), and in the second experiment, we further compared the immunogenicity of live DF-1-tHA with live DF-1-pHA (Figure 4b). The first experiment was conducted in both VALO SPF and Hy-Line Brown chickens, with five animals assigned to each group. In the Hy-Line Brown group, DF-1-based vaccination did not elicit detectable HI or VN antibody titers (Figure S1). Among the VALO SPF chickens, with the exception of one bird inoculated with inactivated DF-1-tHA, only those immunized with live DF-1-tHA exhibited detectable HI and VN antibody titers. HI titers began to rise by the second week post-priming and continued to increase until the third week post-booster, reaching average values of 6.3 ± 1.2 log_2_ at 2 weeks post-booster and average VN titers of 8.9 ± 1.1 log_2_ at 3 weeks post-booster (Figure 4a). The second experiment was conducted exclusively in VALO SPF chickens (Figure 4b), with six animals assigned to each group, comprising five heterozygous and one homozygous individual for the B21 MHC haplotype. Here, DF-1-tHA elicited HI titers comparable to those induced by DF-1-pHA from 1 to 3 weeks post-booster. Although the average VN titer of DF-1-pHA (12.7 ± 1.0 log_2_) was higher than that of DF-1-tHA (8.4 ± 1.2 log_2_) at 3 weeks post-booster, this difference was not statistically significant. No differences in HI and VN titers were observed between homozygous and heterozygous individuals.

### 2.5. Intramuscular Immunization with DF-1 Cells Transiently Expressing M1 or NP Was Sufficient to Induce Virus-Specific Cellular Immune Responses

**Figure 5:**
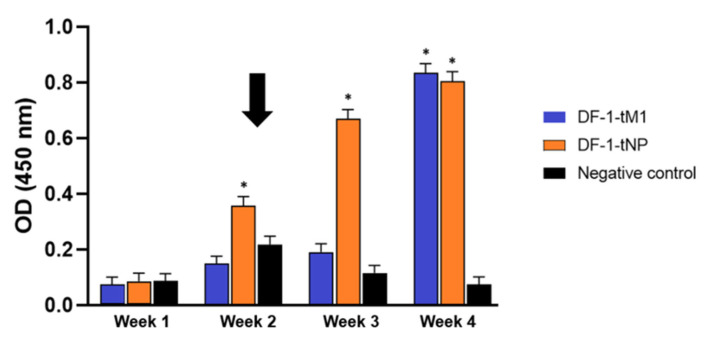
IFN-γ levels in PBMCs across time points post transient DF-1-based M1/NP vaccination. PBMCs were isolated weekly and stimulated with heat-inactivated SL20 virus or without antigen for 48 h. Independent two-sample *t*-tests were performed to assess statistical significance between group means based on five biological replicates (* *p* < 0.05). The tests evaluated whether antigen-stimulated groups differed significantly from the no-antigen control at each time point. The black arrow indicates the booster vaccination time point.

IFN-γ is primarily secreted by Th1 and CD8+ T cells and plays a critical role in antiviral defense and cellular immune responses [14]. To assess vaccine-induced activation, PBMCs were collected weekly from birds immunized with DF-1 cells transiently expressing nucleoprotein (DF-1-tNP) or matrix 1 protein (DF-1-tM1). IFN-γ levels were measured by ELISA after co-stimulation with anti-CD3/CD28 antibodies in the presence or absence of heat-inactivated SL20 (Figure 5).

In the DF-1-tNP vaccine group, stimulation with SL20 induced a sustained and significant increase in IFN-γ levels from 2 to 4 weeks post vaccination, with a clear post-booster enhancement at week 2 (Figure 5) (*p* < 0.05). In contrast, the DF-1-tM1 vaccine group showed a less sustained and temporally inconsistent IFN-γ response, with a statistically significant increase observed only at 4 weeks post vaccination (*p* < 0.05). The negative control group consistently exhibited low absorbance values across all time points.

Animal experiments involving live cells expressing foreign genes delivered via lentiviral vectors are typically required to be conducted in ABSL-2 facilities. However, our finding that transient expression of M1 and NP can also induce immune responses in chickens suggests a potential to lower biosafety facility requirements for future studies and highlights the versatility of this platform for dissecting antigen-specific immunity under less stringent conditions.

### 2.6. CD8+ T Cell Epitopes of HA, M1, and NP Are Present on the Class 1 MHC Molecules of DF-1 Cells

Using the reported binding motif for B21 CD8+ T cell epitopes [15], we identified epitopes in the HA, M1, and NP proteins (Table 1). Specifically, five epitopes were identified in the HA protein, one in the M1 protein, and four in the NP protein. To determine the actual epitopes presented by DF-1 cells expressing viral genes permanently (DF-1-pHA, DF-1-pM1, and DF-1-pNP), we eluted surface peptides from the cells and performed multi-omics high-resolution mass spectrometry. Only peptides exhibiting both *b*-ion and *y*-ion series were considered confirmed CD8+ T cell epitopes (Figure S2). In the case of HA, of the five predicted epitopes, only _395_NKVNSIIDKM_404_ was identified. Subsequent analysis revealed that this epitope is located in the terminal region of helix A in the prefusion state of the HA2 subunit (residues 50–59) and includes the highly conserved residue (K58)-stabilizing HA2 structure [16], as well as a conserved component residue (N53) of the common HA2 epitope [17].

The overlap between the sequences predicted by the T cell epitope-binding motif and those identified through peptide analysis indicates the reliability of the analysis method. This HA epitope is highly conserved among H5Nx IAVs, with 97.6% of isolates exhibiting an identical sequence.

To assess the broader conservation of the predicted NP and M1 epitopes beyond the H5Nx subtype, we conducted sequence homology searches using the NCBI GenBank database. This analysis confirmed that the NP epitope (_212_GRRTRVAYERM_222_) was highly conserved, being present in 98.5% of isolates, whereas the M1 epitope (_133_NRMGTVTAEGA_143_) exhibited a lower conservation rate of 67.9% across multiple influenza A virus lineages (Table 1). These findings are consistent with the generally conserved nature of internal viral proteins.

### 2.7. Multiple CD8+ T Cell Epitope-Binding Motifs in Various Chicken Haplotypes Emphasize the Significance of the NP T Cell Epitope-Binding Region

For NP, the search was extended to identify CD8+ T cell epitope-binding motifs in haplotypes beyond the well-characterized B21 (Table 2). Although these findings do not cover all chicken haplotypes, the presence of multiple predicted binding regions across various haplotypes underscores the relevance of the inferred NP T cell epitope-binding region. Additionally, overlapping sequences were observed between the epitope-binding motifs of B15 and B19 and that of B21. Since B15, B19, and B21 are predicted to present similar peptides, it is reasonable to infer that the level of humoral immunity elicited by these haplotypes in response to DF-1 inoculation would be similar from a self–non-self recognition standpoint.

## 3. Discussion

This study evaluated a host–cell vector platform in which DF-1 chicken fibroblast cells were engineered to express individual influenza viral proteins. Using DF-1 cells expressing HA, we immunized MHC-matched B21 chickens to assess the platform’s capacity to induce antigen-specific immune responses. We demonstrated that live DF-1 cells expressing HA induced strong humoral immunity, comparable to that elicited by DNA vaccines. Moreover, the system enabled the identification of MHC class I-bound CD8+ T cell epitopes directly from host cells using mass spectrometry. These epitopes included highly conserved sequences, such as GRRTRVAYERM from NP, with potential relevance across diverse chicken MHC haplotypes.

The DF-1 cell line was validated as a suitable platform for both transient and stable expression of viral genes. For example, in a previous study, 10-day-old chicks were inoculated twice with 100 µg of pcDNA3.1 vector expressing the HA gene, resulting in HI titers of 2.33 ± 0.82 (6/9 positive) and 5 ± 2.45 (9/9 positive) at 1 and 2 weeks post-booster, respectively [14]. Although there were differences in bird numbers and age compared to our study, the comparable average HI titers (6.28 ± 1.24 and 5.79 ± 1.35 for live DF-1-tHA at 2 weeks post-booster), along with the fact that our approach (using 60 µg per bird) elicited detectable HI titers in all animals after boosting, suggest that our cell-based delivery method induces a more consistent immune response.

Despite sufficient gene expression in DF-1 cells, the inactivation process appeared to prevent these cells from inducing humoral immunity, likely due to their rapid clearance by the immune system. Loss of membrane integrity leads to the release of intracellular DAMPs, which trigger an innate immune response consistent with the “danger hypothesis” [18]. This mechanism may explain why most VALO SPF chickens with the B21 haplotype failed to develop a humoral response following treatment with inactivated HA-expressing DF-1 cells. In contrast, live DF-1-tHA cells also failed to elicit HI titers in Hy-Line Brown chickens, highlighting the importance of MHC haplotype matching [19].

In chickens, chMDA5 induces type I interferon and initiates an immune cascade during IAV infection. Notably, a previous study showed that co-administration of equal amounts (25 µg each) of HA and chMDA5-based vaccines on two occasions induced high HI titers—approximately 9 log_2_—in most chickens (5 out of 6) at 3 weeks post-booster [5]. Applying a similar strategy to our host cell–vector system may reduce the required amount of expression vector while further enhancing humoral immune responses [20].

To further investigate the role of MHC matching in immune responses, a second experiment comparing DF-1-tHA and DF-1-pHA was conducted. Consistent with the previous findings, both heterozygous and homozygous MHC genotypes were present in these experimental groups, yet all animals mounted an immune response following immunization. This outcome can be attributed to the immunogenetic characteristics of chickens, which possess a single dominantly expressed MHC class I molecule. The presence of the B21 haplotype likely facilitated effective antigen presentation and immune activation even in heterozygous individuals.

While HI and VN titers primarily reflect MHC class II–mediated humoral responses, our aim was to demonstrate that the DF-1/B21 platform can simultaneously support antigen-specific antibody induction and facilitate endogenous MHC class I presentation for CD8+ T cell epitope identification. This integrative model enables parallel evaluation of both arms of adaptive immunity within an MHC-defined system. Given the high prevalence of the B21 haplotype in SPF chicken lines and its relevance in experimental vaccinology, the DF-1/B21 model serves as a valuable tool for dissecting immune mechanisms under defined MHC contexts.

Considering the highly variable traits of HA genes in IAVs, the presence of a highly conserved novel CD8+ T cell epitope among H5Nx IAVs was unexpected. Such conservation suggests that these regions are under strong evolutionary constraint, likely because they are critical for viral survival and cannot tolerate mutations even under host immune pressure.

In parallel, VALO SPF chickens have been widely utilized as a model in DNA vaccine protection studies against highly pathogenic H5Nx IAVs. Notably, chickens carrying the B21 haplotype tend to exhibit stronger cross-protective immune responses compared to those lacking this haplotype [4]. Therefore, these results should be interpreted with caution when applied to genetically diverse commercial chicken populations.

In contrast to HA, numerous mutations were identified in M1, likely reflecting host-induced immune evasion mechanisms [21]. The inferred T cell epitope-binding region in M1 is part of a bifunctional membrane–RNA bind, where mutations may not be essential for viral survival [22]. However, the highly conserved CD8+ T cell epitope of NP may be critical for viral survival and, therefore, less prone to variation. Interestingly, it contains the bipartite nuclear localization signal, and mutations R213A, R214A, R216A, E220A, and R221A decreased viral transcription, viral titers, and polymerase function at 33 °C [23].

Building on this finding, the NP epitope identified here (_212_GRRTRVAY_219_) is predicted to be presented not only by the B21 haplotype, but also by B15 and B19 haplotypes in chickens [24], suggesting its broad relevance across avian MHC diversity.

DNA and RNA vaccines undergo transcription and/or translation prior to antigen presentation via both major histocompatibility complex (MHC) class I and II pathways [25]. As a result, they do not directly mimic host antibody–antigen interactions that occur during natural viral infection. In this study, using the VALO SPF–DF-1 cell model, we not only evaluated vaccine efficacy, but also established a system that allows for the direct identification of T cell epitope-binding peptide sequences. This approach enables a closer mimicry of natural viral infection, providing insight into how specific viral proteins interact with the host immune system.

This study suggests that the DF-1/B21 host–cell vector system can serve as a useful model for analyzing immune responses to individual viral antigens under defined MHC conditions. While all experiments were conducted in chickens with the B21 haplotype, this genetically consistent model offers a controlled setting for exploring antigen-specific mechanisms. We observed humoral responses induced by live HA-expressing cells and virus-specific cellular responses from transient expression of internal proteins, with NP-associated responses showing a trend toward sustained IFN-γ production compared to M1. In addition, several candidate MHC class I-bound epitopes were detected from the HA-, NP-, and M1-expressing cell lines, including conserved CD8+ T cell epitopes. These preliminary findings support the potential utility of this platform in antigen evaluation. However, due to the model’s dependence on a single MHC haplotype, caution is warranted in extrapolating the results to genetically diverse or outbred populations. Further studies involving additional MHC haplotypes and functional assays will be required to validate the broader applicability of this system.

## 4. Materials and Methods

### 4.1. Cells, Reagents, and Plasmids

DF-1 cells (ATCC CRL-3586, Manassas, VA, USA) were maintained under standard conditions. Stable DF-1 lines expressing 2.3.4.4b clade H5 HA consensus, Y280-lineage A/chicken/South Korea/SL20/2020 (H9N2) (SL20) M1 or NP were generated via lentiviral transduction (Lenti-X™ Packaging Single Shots, Takara Bio Inc., Shiga, Japan) and selected with 1 μg/mL puromycin for 1 week. MDCK I cells (ATCC CRL-2935, Manassas, VA, USA) were used for virus neutralization assays. The consensus H5 HA gene sequence was generated based on 67 avian influenza virus isolates collected between September and November 2022 (GISAID [26]) (Table S1). This H5 HA gene was cloned into two expression vectors—pcDNA3.1 (Invitrogen, Thermo Fisher Scientific Inc., Waltham, MA, USA) and pLVX-puro (Takara Bio Inc., Shiga, Japan)—using the *Xho*I and *Apa*I restriction sites. The pcDNA3.1 vector was used for transient expression experiments, whereas the pLVX-puro vector was used for the production of stable cell lines. Separately, the M1 and NP genes were cloned into the same vectors using the *Eco*RI and *Apa*I restriction sites (Table S2). For the generation of antiserum, the highly pathogenic H5 HA gene was further modified: the cleavage site sequence (RRKR) was altered to ASGR using splice overlap extension PCR (SOE-PCR). The modified HA gene was then inserted into Hoffmann’s vector and used for reverse genetics [27,28,29]. The resulting reassortant virus (rH5N1) was subsequently propagated and used to produce antiserum for experiments related to HA-specific immune responses.

### 4.2. MHC Genotyping

Peripheral blood mononuclear cells (PBMCs) were isolated from Hy-Line Brown (Yangji Hatchery & Breeding, Pyeongtaek-si, Gyeonggi-do, Republic of Korea) and VALO SPF chickens (VALO BioMedia, Cuxhaven, Germany)) using Lymphoprep™ (Stemcell Technologies Inc., Vancouver, BC, Canada) according to the manufacturer’s protocol. Genomic DNA was extracted using the PURE Cell & Tissue gDNA Extraction Kit (Infusion Tech Inc., Seoul, Republic of Korea) and LEI0258 PCR was performed using published primers and cycling conditions [13].

To confirm the B21 haplotype, sequence-based typing was performed on a subset of samples previously identified as B21-positive by PCR. BF2 locus-specific primers and previously published PCR cycling conditions were used [30] followed by Sanger sequencing (Cosmogenetech, Seoul, Republic of Korea).

To identify heterozygous loci within the BF2 gene region, sequencing chromatograms were analyzed using Mutation Surveyor version 5.2.0 [31]. Forward and reverse reads were aligned, and base calls were manually inspected in regions with overlapping peaks or ambiguous signals. Heterozygous positions were annotated using standard IUPAC nucleotide codes, and only those consistently identified on both strands were retained for further analysis and tabulation.

### 4.3. Vaccine Preparation

DF-1 cells transiently expressing HA, M1, or NP were produced by transfecting 1 × 10^6^ cells per well in six-well plates with 30 µg of DNA using Lipofectamine™ 2000 (Thermo Fisher Scientific, Waltham, MA, USA). Two wells from each plate were used for inoculation per chicken, while stable expression was achieved using Lenti-X™ Packaging Single Shots (Takara Bio Inc., Shiga, Japan) according to the manufacturer’s protocol. For live vaccines, cells together with their Opti-MEM™ supernatant were collected without washing. Inactivated vaccines were prepared by trypsinizing cells (TrypLE™ Express, Thermo Scientific, Waltham, MA, USA), washing, recombining with the collected supernatant, and inactivating with 0.2% formaldehyde under overnight agitation. Successful inactivation was confirmed by trypan blue staining and by seeding a portion of the inactivated preparation onto culture plates to ensure no adherent cell growth.

### 4.4. Confocal Microscopy

DF-1 cells grown on coverslips were fixed with 4% paraformaldehyde (Sigma-Aldrich, St. Louis, MO, USA). The cells were then permeabilized in 0.2% Triton X-100 (Sigma-Aldrich, MO, USA) prepared in PBS containing 2% normal goat serum (Jackson ImmunoResearch Laboratories, West Grove, PA, USA). Finally, they were incubated with a primary antibody (dilution 1:500; derived from VALO SPF chickens immunized with rH5N1 for HA cells or SL20 for NP or M1 cells [32]) and then with Alexa Fluor™ 555-conjugated secondary antibody (dilution 1:1000; Thermo Fisher Scientific Inc., MA, USA). The samples were DAPI-stained (BioLegend, San Diego, CA, USA) and imaged using an Axio Observer Z1/7 microscope equipped with a 63×/1.40 oil immersion objective (Zeiss, Oberkochen, Germany; Zeiss ZEN 3.8 software).

### 4.5. SDS-PAGE and Western Blotting

HA-expressing DF-1 cells (0.3 × 10^6^ cells per well in six-well plates) were cultured in Opti-MEM™. Supernatants (100 μL) were collected on days 1, 3, 5, and 7, stored at −70 °C, and analyzed using the same method as described in previous studies [33]. For comparative analysis, cell lysates were also prepared using RIPA lysis buffer following the manufacturer’s instructions (Thermo Fisher Scientific Inc., Waltham, MA, USA). The primary antibody was derived from VALO SPF chicken serum immunized with PR8-backbone H5 HA, and Anti-chicken IgG-HRP (Bethyl Laboratories Inc., Montgomery, TX, USA) was used as the secondary antibody. As a positive control, β-actin was used as a loading control for cell lysates. After protein transfer, membranes were incubated with a chicken-reactive anti-β-actin monoclonal antibody (clone C4; Santa Cruz Biotechnology, Dallas, TX, USA) as the primary antibody, followed by incubation with an HRP-conjugated goat anti-mouse IgG secondary antibody (Abcam, Cambridge, UK), both at 1000 times dilution.

### 4.6. Vaccination and Sampling

Three-week-old female Hy-Line Brown (non-SPF, obtained from Yangji Hatchery & Breeding, Pyeongtaek-si, Gyeonggi-do Republic of Korea) and VALO SPF chickens (SPF, obtained from VALO BioMedia, Cuxhaven, Germany) were assigned to experimental groups and housed under biosafety level 2 (BSL-2) conditions with ad libitum access to food and water and a 12 h light/dark cycle. Chickens were assigned to experimental groups and immunized intramuscularly with one of the formulations described below, with each dose comprising 2 × 10^6^ HA expressing cells per chicken. In the initial experiment, groups consisted of five birds each and received one of the following formulations: (i) live DF-1 cells transiently expressing HA (DF-1-tHA), (ii) inactivated DF-1 cells transiently expressing HA, or (iii) inactivated DF-1 cells stably expressing HA (DF-1-pHA).

In a subsequent comparison study conducted exclusively in VALO SPF chickens, groups consisted of six birds each, with each group including one B21 homozygote and five heterozygotes. These groups were immunized with either live DF-1-tHA or DF-1-pHA.

Serum samples were collected weekly, and booster immunizations were administered 2 weeks after the primary vaccination.

In a parallel study to evaluate cellular immune responses, VALO SPF chickens were immunized with DF-1 cells transiently expressing either the NP (DF-1-tNP) or M1 (DF-1-tM1) gene. Each group consisted of five B21-haplotype birds and received 2 × 10^6^ antigen-expressing DF-1 cells intramuscularly, following the same prime–boost schedule as used for DF-1-tHA. Weekly blood samples were collected for 5 weeks post-vaccination for peripheral blood mononuclear cells (PBMC) isolation using Lymphoprep (Stemcell Technologies Inc., Vancouver, BC, Canada). Three weeks after the booster, PBMCs were isolated to evaluate immune responses via IFN-γ ELISA.

### 4.7. Hemagglutination Inhibition (HI) and Virus Neutralization (VN) Assays

HI assays were performed on RDE-treated serum [34] and PR8-backbone H5 HA virus. For VN, PR8-backbone H5 HA virus was incubated with VALO SPF serum and applied to MDCK-I cells; after 3 days, HA titers in the supernatant were determined.

### 4.8. ELISA for IFN-γ Detection

PBMCs (0.3 × 10^6^/well) were stimulated in 12-well plates with purified, heat-inactivated SL20 virus at a final concentration of 10 μg/mL (56 °C for 1 h post sucrose gradient) [15]. CD3 pre-coating and soluble CD28 were used for co-stimulation, using Mouse Anti-Chicken CD3-UNLB and Mouse Anti-Chicken CD28-UNLB antibodies (both from SouthernBiotech, Birmingham, AL, USA). A no-stimulation control (medium only) was included in all assays. Supernatants were collected 48 h post-stimulation, and IFN-γ quantified using the Chicken IFN-γ CytoSet ELISA kit (Invitrogen, Waltham, MA, USA). Five replicates per condition were analyzed. Statistical analyses were performed using IBM SPSS Statistics for Windows, version 26.0 (IBM Corp., Armonk, NY, USA). Group means were compared using Welch’s *t*-test with Bonferroni correction; *p* < 0.05 was considered statistically significant.

### 4.9. Multi-Omics Mass Spectrometry for CD8+ T Cell Epitope Mapping

T cell epitopes were isolated with minor adaptations from a previously reported protocol [35]. Briefly, DF-1 cells expressing HA, M1, or NP genes were treated with citrate–phosphate buffer (pH 3.3) to elute surface-bound peptides, followed by peptide purification using a SepPak C18 cartridge (Waters Corporation, Milford, MA, USA) and 60% acetonitrile. Purified peptides were dried using a BUCHI Rotavapor™ R-300 rotary evaporator (BUCHI Labortechnik AG, Flawil, Switzerland), resuspended in citrate–phosphate buffer, and subsequently analyzed using multi-omics high-resolution mass spectrometry (Orbitrap Exploris 240 mass spectrometer (Thermo Fisher Scientific Inc., Waltham, MA, USA)). Peptide identifications were based on MS/MS fragmentation. Fragment ion assignments were made using *b-* and *y*-ion series, and peptide–spectrum matches were confirmed through manual validation of representative spectra (Figure S2).

### 4.10. Sequence Variation and MHC-I Binding Predictions

HA epitope sequences (complete CDS; 567 amino acids in length) from 23,647 HPAI H5 isolates (GISAID, 1905–31 October 2024) were analyzed. For M1 and NP, predicted T cell epitopes were identified via BlastP against NCBI GenBank (100% query cover; maximum targets: 1000 for M1, 5000 for NP), retaining sequences with single amino acid differences, excluding mixed influenza types.

## Figures and Tables

**Table 1:** Predicted and identified CD8+ T cell epitopes present on the B21 haplotype MHC class 1 molecules of DF-1 cell surface.

Protein	Predicted Epitope ^a^	Identified Epitope (Percentage)	Variation (%)
HA	_23_Y**H**ANNST**E**Q**V**_32_	_395_N**K**VNSII**D**K**M**_404_ ^b,c^ (97.6)	Others (2.4)
	_402_D**K**MNTQF**E**A**V**_411_		
	_487_H**K**CDNECM**E**S**V**_497_		
	_395_N**K**VNSII**D**K**M**_404_		
	_465_D**K**VRLQLR**D**N**A**_475_		
M1	_133_N**R**MGTVTA**E**G**A**_143_	_133_N**R**MGTVTA**E**G**A**_143_ ^b^ (67.9)	N**R**MGTVTA**E**V**A** (14.3)
			N**R**MGTVTT**E**G**A** (12.2)
			N**R**MGTVTA**E**A**A** (2.1)
			Other (3.5)
NP	_435_G**R**TSDMRT**E**I**I**_445_	_212_G**R**RTRVAY**E**R**M**_222_ ^b^ (98.5)	G**R**RTRIAY**E**R**M** (1.5)
	_99_R**R**DGKWMR**E**L**I**_109_		
	_120_W**R**QANKG**E**D**A**_129_		
	_212_G**R**RTRVAY**E**R**M**_222_		

^a^ The anchoring amino acid residues of B21 haplotype binding CD8+ T cell epitope motif (X-**H/K/R/E**-X-X-X-X-X-(X)-**E/D/L**-X-**A/V/L/I/F/M**) are represented with bold font, while variations are underlined. ^b^ Epitope sequences with both *b-ion* and y*-ion* on the MS peak figure. ^c^ Data derived from the GISAID database.

**Table 2:** CD8+ T cell epitope-binding motifs in haplotypes other than the known B21 MHC haplotype.

Haplotypes (Binding Motif)			
B4 X-D/E-X-X-D/E-X-X-(X)-E/L/I	B12 X-X-X-X-V/I-X-X-(X)-V/L/I	B15 X-R-X-X-X-X-X-(X)-Y	B19 X-R-X-X-X-X-X-Y/P/L/F
_10_YEQMETGGE_18_	_21_NATEIRASV_29_	_212_GRRTRVAY_219_ *	_64_ERMVLSAF_71_
_111_YDKEEIRRI_119_	_25_IRASVGRMV_33_		_76_NRYLEEHP_83_
_251_AEIEDLIFL_259_	_29_VGRMVSGI_36_		_212_GRRTRVAY_219_ *
_371_METMDSNTL_379_	_53_EGRLIQNSI_61_		_347_IRGTRMVP_354_
	_59_NSITIERMV_67_		_451_ARPEDVSF_458_
	_112_DKEEIRRI_119_		
	_179_AGAAVKGI_186_		
	_182_AVKGIGTMV_190_		
	_186_IGTMVMEL_193_		
	_186_IGTMVMELI_194_		
	_190_VMELIRMI_197_		
	_193_LIRMIKRGI_201_		
	_235_QRAMVDQV_242_		
	_249_GNAEIEDL_256_		
	_249_GNAEIEDLI_257_		
	_276_LPACVYGL_283_		
	_308_QNSQVFSL_315_		
	_308_QNSQVFSLI_316_		
	_339_EDLRVSSFI_347_		
	_410_PTFSVQRNL_418_		
	_459_QGRGVFEL_466_		

* indicates T cell epitope-binding motifs that are also present in the B21 haplotype.

## Data Availability

The data presented in this study are available in this article and the Supplementary Materials.

## Supplementary Materials

The following supporting information can be downloaded at https://www.mdpi.com/article/10.3390/ijms26157492/s1.

## Abbreviations

The following abbreviations are used in this manuscript:

IAV	Influenza A virus
SPF	Specific pathogen-free
MHC	Major histocompatibility complex
HA	Hemagglutinin
M1	Matrix 1
NP	Nucleocapsid protein
PBMC	Peripheral blood mononuclear cells
HI	Hemagglutination inhibition
VN	Virus neutralization
DF-1-tHA	Live DF-1 cells transiently expressing HA
DF-1-pHA	DF-1 cells permanently expressing HA
DF-1-pM1	DF-1 cells permanently expressing M1
DF-1-pNP	DF-1 cells permanently expressing NP
