# Supplementary material for: A Host Cell Vector Model for Analyzing Viral Protective Antigens and Host Immunity
Source: Int J Mol Sci. 2025 Aug 2;26(15):7492. doi: 10.3390/ijms26157492 (PMC12347372; doi:10.3390/ijms26157492)
Supplement: Supplementary file 1 [file ijms-26-07492-s001.zip › supplement.pdf]

Table S1. Strains from the GISAID database used for HA consensus configuration

GISAID ID	Isolated species	Isolated country	Isolation date (Year-Month-Date)
114595	Chicken	England	2022-09-02
27	Common tern	Netherlands	2022-09-02
16	Greylag goose	Netherlands	2022-09-02
116603	chicken	Wales	2022-09-04
4	Lesser blacked-backed gull	Netherlands	2022-09-04
117887	Domestic duck	England	2022-09-09
18	European herring gull	Netherlands	2022-09-10
9	Mallard	Netherlands	2022-09-11
22VIR10586-6	Northern gannet	Spain	2022-09-15
17	Greylag goose	Netherlands	2022-09-16
10918 0002	Anser anser domesticus	Belgium	2022-09-16
10906 0006	Pavo	Belgium	2022-09-18
2	Mute swan	Netherlands	2022-09-19
22VIR10586-4	laying hen	Spain	2022-09-19
H397-N	Domestic goose	Poland	2022-09-20
19	European herring gull	Netherlands	2022-09-21
11304 0009	Cygnus olor	Belgium	2022-09-21
22VIR9126-7	Waiian goose	Italy	2022-09-23
11304 0003	Ardea cinere	Belgium	2022-09-23
1409C001T1	Peregrine Falcon	Japan (Kanagawa)	2022-09-25
19	Barnacle goose	Netherlands	2022-09-26
11248 0001	Chicken	Belgium	2022-09-26
123975	Chicken	England	2022-09-28
4	Eurasian curlew	Netherlands	2022-09-28
117298	Mute swan	England	2022-09-30
18	Mallard	Netherlands	2022-10-01
20	European herring gull	Netherlands	2022-10-02
11599 0005	Anas platyrhynchos domesticus	Belgium	2022-10-04
0410D001	White-fronted goose	Japan (Miyagi)	2022-10-04
Q71	Eurasian wigeon	Japan (Hokkaido)	2022-10-08
2	Sanderling	Netherlands	2022-10-09
4	Great black-backed gull	Netherlands	2022-10-09
22VIR9673-3	Goose	Italy	2022-10-09
11943 0003	Chicken	Belgium	2022-10-10
22	Mallard	Netherlands	2022-10-13
22VIR9832-3	chicken	Italy	2022-10-17
24	Mallard	Netherlands	2022-10-18
22VIR9917-52	teal	Italy	2022-10-19

22VIR10097-4	Swan	Italy	2022-10-21
22VIR10097-3	Swan	Italy	2022-10-21
3	Common teal	Netherlands	2022-10-22
12395 0002	Chicken	Belgium	2022-10-24
12318 0001	Mixed domestic anatidae	Belgium	2022-10-24
12102 0001	Mixed domestic psianidae	Belgium	2022-10-24
26	Mallard	Netherlands	2022-10-25
22VIR10267-7	laying hen	Italy	2022-10-25
2	Eurasian wigeon	Netherlands	2022-10-29
2	Common buzzard	Netherlands	2022-10-31
22VIR10534-3	Rural duck	Italy	2022-10-31
4	Eurasian wigeon	Netherlands	2022-11-01
22VIR10651-4	Rural chicken	Italy	2022-11-02
22VIR10627-4	Turkey	Italy	2022-11-02
22VIR10576-13	teal	Italy	2022-11-02
22VIR10789-2	Rural goose	Italy	2022-11-04
22VIR10737-10	Swan	Italy	2022-11-04
22VIR10733-4	Turkey	Italy	2022-11-04
22P023203	chicken	France	2022-11-08
22VIR10996-6	Rural laying hen	Italy	2022-11-08
2	Gadwall	Netherlands	2022-11-09
6	Common teal	Netherlands	2022-11-09
22VIR10995-7	Swan	Italy	2022-11-09
11	Black-headed gull	Netherlands	2022-11-11
1	Great Cormorant	Netherlands	2022-11-11
22VIR11262-1	Turkey	Italy	2022-11-14
152473	Domestic duck	England	2022-11-15
22VIR11246-6	Chicken	Italy	2022-11-15
K22-730-1	Spot-billed duck	Korea	2022-11-17

Table S2. Primers used in this study

Usage	Name	Sequence (5'-3')	Melting temperature (°C)
Initial PCR from synthetic products (5' end)	(1)synHA-F	ATGGAGAACATAGTACTTCTCCTTGC	57
	(1)synHA-R	TAGAGGACTATTTCTGAGCCC	52
Initial PCR from synthetic products (3'' end)	(2)synHA-F	GCATCAGGTAGGGGCC	51
	(2)synHA-R	ACAATGCAGAATTTGCATTTAA	50
SOE PCR for ASGR (attenuated) sequence	ASGR_syn1+syn2_SOE_F	CAGAAATAGTCCTCTAGCATCAGGTAGGGGCCTATTTGGGGCGATAGCAGGGTTTA	78
	ASGR_syn1+syn2_SOE_R	TAAACCCTGCTATCGCCCCAAATAGGCCCCTACCTGATGCTAGAGGACTATTTCTG	78
SOE PCR for consensus sequence	HA_syn1+syn2_SOE_F	CAGAAATAGTCCTCTAAGAGAAAGGAGAAGAAAAAGAGGC CTATTTGGGGCGATAGCAGGGTTTA	77
	HA_syn1+syn2_SOE_R	CTCTTTTTCTTCTCCTTTCTCTTAGAGGACTATTTCTGAGCCCAGTCGCAAGGACCAACT	77
Restriction enzyme addition	XhoI_F	TCGACTCGAGACCATGGGTAGCAAAAGCAGGGGTTCACTCTGTCAAAA	72
	ApaI_R	TCGAGGGCCCAGTAGAAACAAGGGTGTTTTTAACTACAATTAGAGCTCATAAATTT	68
Experimental cell-line development expressing the M1 gene	EcoRI_M1_F	TCGAGAATTCACCATGGGTATGAGCCTTCTAACCGAGGTCGAAACGT	71
	ApaI_M1_R	TCGAGGGCCCTCACTTGAACCGCTGCAGCTG	73
Experimental cell-line development expressing the NP gene	EcoRI_NP_F	TCGAGAATTCACCATGGGTATGGCGTCTCAAGGCACCAAACGA	72
	ApaI_NP_R	TCGAGGGCCCTCAACTGTCATATTCCTCTGCATTGTCTCC	70
