# Supplementary figures and images for: A Host Cell Vector Model for Analyzing Viral Protective Antigens and Host Immunity
Source: Int J Mol Sci. 2025 Aug 2;26(15):7492. doi: 10.3390/ijms26157492 (PMC12347372; doi:10.3390/ijms26157492)

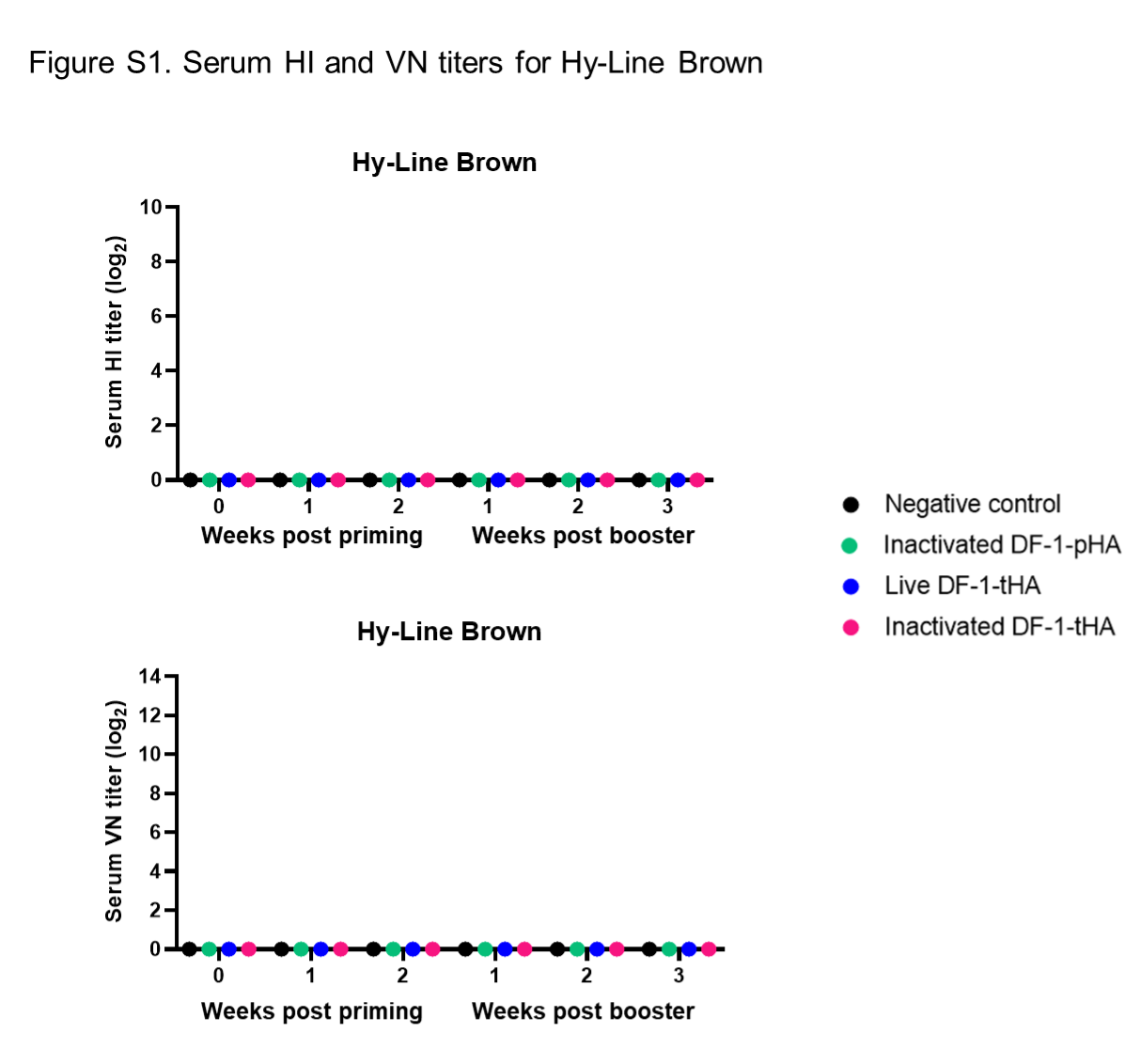

Supplement: Supplementary file 1 [file ijms-26-07492-s001.zip › Figure S1.png]

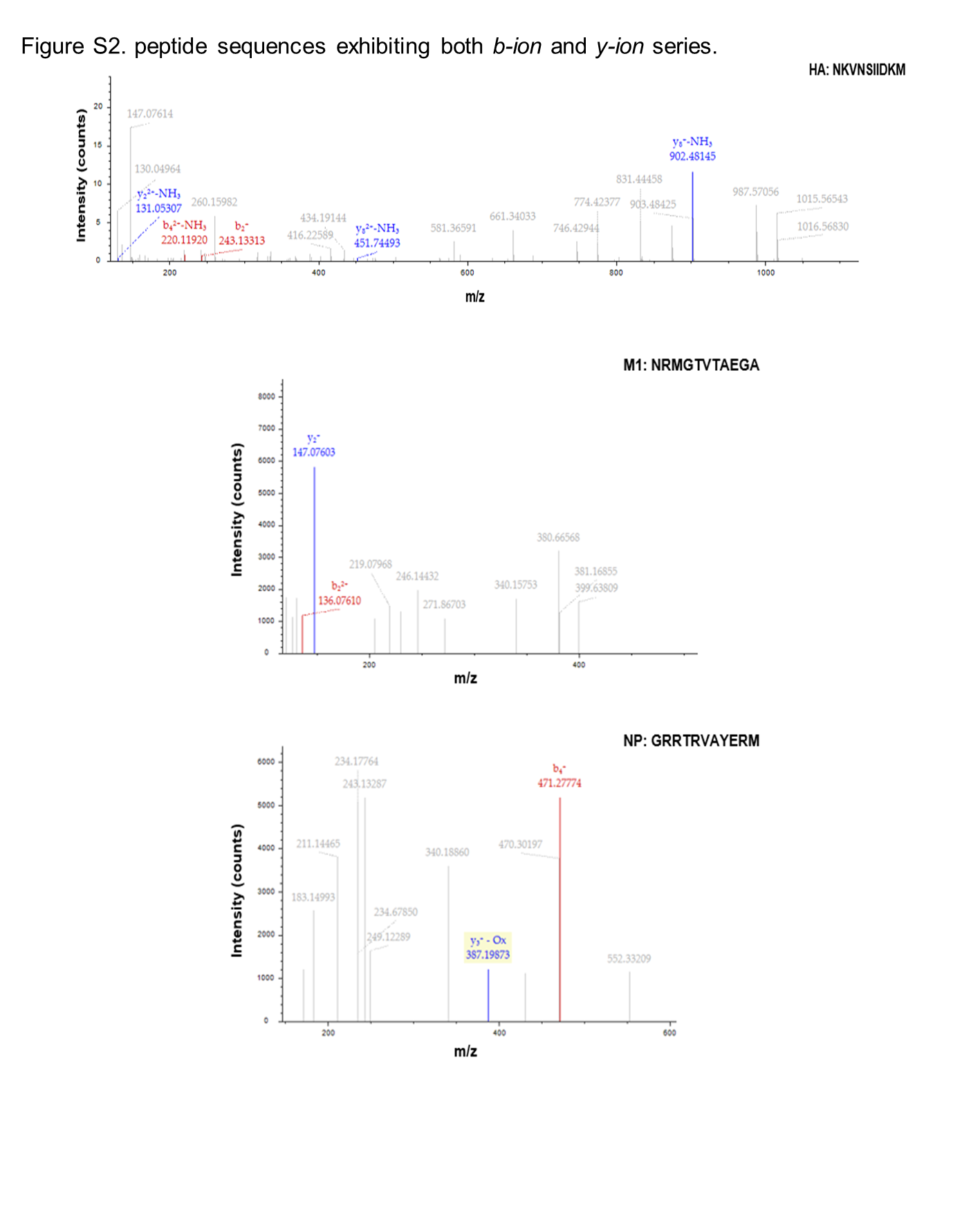

Supplement: Supplementary file 1 [file ijms-26-07492-s001.zip › Figure S2.png]
